# Is virtue its own reward? Moral identity, empathy, and volunteering during adolescence as predictors of subsequent epigenetic aging

**DOI:** 10.1111/aphw.70026

**Published:** 2025-04-05

**Authors:** Carlos N. Espinoza, Marlon Goering, Alison E. Dahlman, Amit Patki, Hemant K. Tiwari, Caroline G. Richter, Sylvie Mrug

**Affiliations:** ^1^ Department of Psychology University of Alabama at Birmingham Birmingham USA; ^2^ Department of Psychology Georgetown University Washington USA; ^3^ Department of Psychology University of South Carolina Columbia USA; ^4^ Department of Biostatistics University of Alabama at Birmingham Birmingham USA

**Keywords:** empathy, epigenetic aging, moral identity, psychological well‐being, self‐esteem, volunteering

## Abstract

Higher levels of moral identity, empathy, and volunteering (virtues) are associated with increased self‐esteem and psychological well‐being, which, in turn, are predictive of fewer health problems. Epigenetic aging, a marker of health, reflects the rate at which individuals age biologically relative to their chronological age. Epigenetic aging is shaped by behavioral factors and environmental stressors, but the effects of moral identity, empathy, and volunteering on epigenetic aging are underexplored. Thus, this study examined if these three dimensions of virtue predict epigenetic aging during adolescence and if these relationships are mediated by self‐esteem and psychological well‐being. The sample included 1,213 adolescents (51% female; 62% Black, 34% Non‐Hispanic White, 4% Other race/ethnicity) that participated at three time points between 2004 and 2017 (*M*age 13, 16, 19 years). Results revealed that higher moral identity and empathy were associated with higher self‐esteem and psychological well‐being during early adolescence. Moreover, higher empathy during early adolescence was associated with slower epigenetic aging on the GrimAge clock during late adolescence. Path analyses adjusting for covariates showed that higher self‐esteem during middle adolescence predicted slower epigenetic aging in late adolescence, but none of the three virtues in early adolescence predicted self‐esteem, psychological well‐being, or epigenetic aging over time.

## INTRODUCTION

The Roman statesman and philosopher Cicero stated that “virtue is its own reward”, meaning that striving to live up to moral principles should be rewarding in itself (Cicero, ca. 45 BC/Parler, 2018). While Cicero admirably advocated for doing good purely for its own sake, being virtuous may bring additional rewards. Indeed, empirical research has linked virtuous cognitions and behaviors, such as moral identity, empathy, and volunteering, to better psychological well‐being and self‐esteem (Choi et al., 2016; Goering, Espinoza, et al., 2024; Hardy et al., 2013; Piliavin & Siegl, 2007; Stuart et al., 2020; Supervía et al., 2023). In turn, psychological well‐being and self‐esteem are predictive of better health across the lifespan (Orth et al., 2012; Stinson et al., 2008). An early marker of later chronic health problems is a faster rate of biological aging, which can be measured through DNA methylation‐based epigenetic clocks and is referred to as epigenetic aging (Levine et al., 2018; Palma‐Gudiel et al., 2022).

A critical developmental period for the development of moral identity, empathy, and volunteering is adolescence (Krettenauer & Hertz, 2015; U. S Census Bureau, 2021; Van der Graaff et al., 2014). However, little research has explored the potential effects of these virtues on epigenetic aging during this time. It is also not clear whether these relationships may be mediated by the positive psychological outcomes previously associated with these virtues. Thus, this study will explore moral identity, empathy, and volunteering in early adolescence as predictors of epigenetic aging in late adolescence, and whether these relationships are mediated by higher self‐esteem and psychological well‐being in middle adolescence.

## VIRTUES AND HEALTH

A virtue is a tendency of an individual to seek and engage in acts of beneficence, which is then reflected in cognitions, emotions, and prosocial behaviors (Baumeister & Exline, 1999; Simmons, 2014). While many virtues exist, moral identity, empathy, and volunteering were selected as a focus of this study because they together offer a comprehensive and multidimensional assessment of virtue, spanning the domains of thought, feeling, and action. Moral identity represents the cognitive dimension of virtue, as it reflects the degree to which moral principles such as fairness, compassion, and honesty are central to one's self‐concept (Aquino & Reed, 2002). Empathy captures the emotional dimension, encompassing both cognitive and affective processes that enable individuals to understand and share others' emotions, which is a crucial precursor to prosocial action (Singer, 2006). Finally, volunteering represents the behavioral dimension, as it involves an intentional, unpaid commitment to helping others beyond one's immediate social circle (Salamon & Sokolowski, 2016). By selecting these three constructs, this framework ensures a balanced evaluation of virtue—encompassing not only how individuals think about morality (moral identity), how they feel towards others (empathy), but also how these internal processes translate into real‐world prosocial behavior (volunteering). This tripartite approach provides a more holistic measure of virtue than any single dimension alone.

Adolescence is a critical developmental period for cultivating virtues (Krettenauer & Hertz, 2015; Moorfoot et al., 2015; Van der Graaff et al., 2014). While virtues facilitate prosocial behaviors that benefit others (Ding & Lu, 2016; Hertz & Krettenauer, 2016), developing virtues such as high moral identity, empathy, and engaging in volunteer work can also yield personal health benefits. For example, moral identity is associated with fewer negative health behaviors in college students (Hardy et al., 2013) and volunteering has been linked to lower cholesterol, inflammation, and body mass index in adolescents (Schreier et al., 2013). Moreover, empathy may confer health benefits, as general virtuous cognitions have been linked to better self‐reported health in adulthood (Hill et al., 2013) and higher empathy levels specifically have been associated with lower levels of inflammation in adolescence (Schreier et al., 2013).

Therefore, it is possible that moral identity, empathy, and volunteering may also be related to other health markers such as a slower rate of biological aging. Biological aging can be measured with epigenetic “clocks” (e.g., GrimAge, DunedinPACE, and PhenoAge), which estimate biological age and the risk for disease and mortality from DNA methylation patterns on CpG sites across the genome (Lin et al., 2016). Previous research has linked virtuous cognitions and affections such as being compassionate with slower epigenetic aging among young adults (Dobewall et al., 2023). Similarly, high levels of empathy during adolescence have been associated with slower epigenetic aging in young adulthood (Goering, Moore, et al., 2024).

One way that virtues may be linked to reduced epigenetic aging is by lowering stress responses. For example, moral identity, empathy, and volunteering may reduce stress via engagement in prosocial behavior that may foster positive emotions, which in turn help mitigate stress (Ferguson et al., 2021; Folkman, 2008). Such prosocial behavior may also facilitate positive responses and support from others, which may help alleviate stress (Auerbach et al., 2011; Boele et al., 2019). Ultimately, this reduced time under stress may contribute to a slower rate of epigenetic aging (Harvanek et al., 2021). However, no study has comprehensively investigated multiple dimensions of virtue in relation to epigenetic aging during adolescence. This study seeks to fill this gap by investigating moral identity, empathy, and volunteering as predictors of epigenetic aging in adolescence.

## THE MEDIATING ROLES OF SELF‐ESTEEM AND PSYCHOLOGICAL WELL‐BEING

The potential effects of moral identity, empathy, and volunteering on slower epigenetic aging may be mediated by higher self‐esteem and psychological well‐being. Self‐esteem is defined as confidence and satisfaction with oneself (Zeigler‐Hill et al., 2016), while psychological well‐being refers to an individual's positive emotional and mental state (Hernandez et al., 2018). Both self‐esteem and psychological well‐being have been linked with virtuous cognitions and behaviors (Brown et al., 2012; Hardy et al., 2013; Hui et al., 2020; Supervía et al., 2023; Vinayak & Judge, 2018).

Moral identity may foster self‐esteem and well‐being through the processes of identity exploration and achievement, which are crucial developmental tasks during adolescence (Meeus et al., 2010). As individuals internalize moral values into their sense of self, they may experience greater self‐worth and coherence, reinforcing psychological well‐being. Indeed, research has linked identity achievement to self‐esteem (Ryeng et al., 2013) and psychological well‐being (Waterman, 2007). However, little research has explored the prospective relationship of moral identity with self‐esteem and psychological well‐being. A recent meta‐analysis found no studies that examined these relationships longitudinally (Goering, Espinoza, et al., 2024), highlighting a critical gap. Thus, a key contribution of this study is to clarify whether moral identity in early adolescence fosters self‐esteem and psychological well‐being in later adolescence, providing empirical support for its developmental significance.

By contrast, empathy and volunteering are more firmly established as predictors of self‐esteem and psychological well‐being (Bowman et al., 2010; Fu et al., 2017; Green et al., 2018; Vinayak & Judge, 2018). These effects may be driven by the intrinsic satisfaction and external social reinforcement that accompany prosocial engagement. Specifically, engaging in empathetic and prosocial behaviors may enhance one's sense of social belonging and competence, reinforcing positive self‐perceptions (Ferguson et al., 2021). Additionally, a meta‐analysis linked higher empathy to greater social support from peers in adolescence (Boele et al., 2019), which may in turn contribute to self‐esteem and psychological well‐being (Butler et al., 2022; Colarossi & Eccles, 2003). Because adolescence is a period of heightened sensitivity to social interactions, the emotional rewards and social reinforcement derived from prosocial behaviors may be particularly influential in shaping self‐esteem and psychological well‐being during this stage.

Although research on self‐esteem, psychological well‐being, and epigenetic aging in adolescence is still emerging, existing evidence suggests that these psychological factors contribute to better health outcomes, which in turn may slow epigenetic aging. Self‐esteem and psychological well‐being have been linked to better physical health in adolescence (Orth et al., 2012; Stinson et al., 2008), which is related to slower epigenetic aging (Levine et al., 2018; Palma‐Gudiel et al., 2022). The stress‐buffering effects of self‐esteem and psychological well‐being may be a key mechanism driving this relationship, as individuals with greater self‐confidence and emotional resilience experience lower physiological stress responses, which in turn contribute to healthier biological aging trajectories (Dockray & Steptoe, 2010; Piekarska, 2020). Moreover, it has been proposed that social–emotional experiences may affect health through DNA methylation, the basis of epigenetic aging, which alters gene expression (Cunliffe, 2016; Hao et al., 2018). Because adolescence is a sensitive period for social–emotional development, self‐esteem, and psychological well‐being may serve as crucial pathways through which social experiences shape long‐term biological health outcomes. Given that self‐esteem and psychological well‐being are particularly responsive to social–emotional processes such as social stress and social acceptance during adolescence (Birkeland et al., 2014; Ditzfeld & Showers, 2013; Plenty & Mood, 2016; Schwarz et al., 2012), they may serve as key mechanisms linking moral identity, empathy, and volunteering to epigenetic aging.

In summary, this study proposes that self‐esteem and psychological well‐being mediate the relationship between virtues and epigenetic aging by fostering positive psychological states and reducing stress‐related biological aging. Specifically, the virtues of moral identity, empathy, and volunteering may enhance self‐esteem and psychological well‐being by promoting self‐worth, social belonging, and emotional satisfaction. These enhanced psychological states, in turn, may buffer against stress‐related biological processes that accelerate aging. By investigating this pathway, this study will provide novel insights into how adolescent virtues shape long‐term biological health outcomes, advancing our understanding of the psychological and physiological benefits of virtue development.

## CURRENT STUDY

The current study aimed to examine the relationships of moral identity, empathy, and volunteering with epigenetic aging, as well as self‐esteem and psychological well‐being as mediators of these links during adolescence. It was hypothesized that moral identity, empathy, and volunteering in early adolescence would predict slower epigenetic aging in late adolescence. It was also hypothesized that these prospective effects of moral identity, empathy, and volunteering on slower epigenetic aging would be mediated by higher self‐esteem and psychological well‐being in middle adolescence.

## METHODS

### Participants

This study involved 1,213 adolescents (51% female, 49% male; 62% Black, 34% Non‐Hispanic White, 4% Other race/ethnicity) who participated in Waves 2, 3, and 4 of the longitudinal Healthy Passages Study at the Birmingham site between 2004 and 2017. The initial sample at Wave 1 involved 1,597 adolescents in fifth grade who were recruited from public schools in Birmingham, Alabama, USA. Follow‐up interviews were conducted during seventh grade (Wave 2, N = 1,535, mean age = 13.10 years), 10th grade (Wave 3, N = 1,392, mean age = 16.1 years), and during late adolescence and emerging adulthood (Wave 4, N = 1,273, mean age = 19.7 years). This study used data from Waves 2, 3, and 4 and only included youth who had epigenetic data at Wave 4 (analytic N = 1,213). Throughout the manuscript, the three time points are referred to as Time 1 (Wave 2), Time 2 (Wave 3), and Time 3 (Wave 4).

### Procedures

At Waves 1–3, adolescents participated together with their caregivers, and both completed private interviews conducted by trained staff. At Wave 4, interviews were conducted only with adolescents who were interviewed, and who also provided saliva samples for DNA methylation analyses using Oragene DNA OG‐500 kits. To ensure sample integrity, participants did not consume any food or drink for at least 30 minutes before sample collection. Additionally, adolescents' height and weight were measured using a portable stadiometer and electronic weight scale. At each wave, informed consent was obtained from adult participants and caregivers of minors, while participants under the age of 18 provided informed assent. All measures and procedures were approved by the university's institutional review board.

### Measures

#### Moral identity

Moral identity was assessed at Time 1 through youth self‐report using an adapted version of the Search Institute Profiles of Student Life Attitudes and Behavior Survey's Values Subscale (Search Institute, 1996). Adolescents were asked to rate the importance of six moral values ‐ helpfulness, fairness, honesty, integrity, altruism, and work ethic, corresponding to values included in the commonly used Self‐Importance of Moral Identity Scale (Aquino & Reed, 2002). Adolescents rated the importance of each value on a five‐point scale ranging from *Not Important* (1) to *Extremely Important* (5). An example item is “How important is it to you in your life to help to make sure that all people are treated fairly?” Responses were averaged, with higher scores indicating a stronger moral identity (Cronbach's α = .87).

#### Empathy

Empathy was assessed at Time 1 via youth self‐report using the Social–Emotional Competency Empathy Scale (Demaray et al., 1995). Adolescents responded to 10 items about understanding how other adolescents and adults feel, as well as sympathetic emotional responding, using a three‐point scale ranging from *Never* (1) to *Very often* (3). An example item is “How often do you feel sorry for others when bad things happen to them?”. Responses were averaged with higher scores indicating more empathy (Cronbach's α = .85).

#### Volunteering

Volunteering was assessed at Time 1 by the primary caregiver's report using a single item that asked how many times per week their child engages in volunteer work. From these responses, a dichotomous item was created indicating whether the adolescent engaged in any volunteering (1) or not (0). A similar procedure has been used in prior studies (Lanza et al., 2023).

#### Self‐esteem

Self‐esteem was assessed at Times 1 and 2 via youth report using the six‐item Global Self‐Worth subscale from the Self‐Perception Profile for Children (Harter, 2012). An example item is “Some kids are often unhappy with themselves; other kids are pretty pleased with themselves. Which statement best describes you?”. A follow‐up question asks whether the prior statement is “sort of true” or “really true”. Based on these responses the individual items are scored on a four‐point scale (1 – low self‐esteem and really true, 2 – low self‐esteem and sort of true, 3 – high self‐esteem and sort of true, 4 – high self‐esteem and really true). The scores from the six items were averaged with higher values indicating greater self‐esteem (Cronbach's α = .73 and .74 at Times 1 and 2).

#### Psychological well‐being

Psychological well‐being was assessed at Times 1 and 2 through youth self‐report using four items from the Emotional Functioning subscale of the Pediatric Quality of Life Inventory (Varni et al., 2001). Items were rated on a five‐point scale ranging from *Almost always* (1) to *Never* (5). An example item is “You feel afraid or scared”. The individual responses were coded so that higher scores indicated more psychological well‐being and averaged (Cronbach's α = .73 and .70 at Times 1 and 2).

#### Epigenetic aging

Epigenetic aging was computed from salivary DNA collected at Time 3. From the collected samples, DNAs were extracted using the pure‐gene extraction method (Mrug et al., 2024). Methylation analyses on the DNA samples were then performed with the Illumina Infinium MethylationEPIC BeadChip genome‐wide methylation analysis tool (Dhingra et al., 2019). Probe and sample level quality controls were conducted in the R‐package Minfi including within array normalization, batch/plate/chip adjustment, chemistry correction, and background correction. Methylation was quantified in β‐values, which describe the level of methylated fluorescent intensity adjusted for overall intensity (Bibikova et al., 2006).

Consistent with recommendations for studies on epigenetic aging (Duan et al., 2022), this study employed three different epigenetic aging clocks ‐ GrimAge, DunedinPACE, and PhenoAge, to assess biological aging based on DNA methylation patterns. GrimAge scores were computed from DNA methylation on 1,030 CpG sites using a publicly available epigenetic age calculator (https://dnamage.genetics.ucla.edu/). The DunedinPACE scores were computed from the DNA methylation pattern on 173 CpG sites (Belsky et al., 2022). Finally, PhenoAge scores were computed from the DNA methylation pattern on 513 CpG sites (Levine et al., 2018). Scores on each epigenetic clock were regressed on chronological age at Time 3 and the resulting residuals were used as indicators of accelerated epigenetic aging.

#### Covariates

Covariates included sociodemographic characteristics (age, sex, race/ethnicity, household income) and standard confounders related to epigenetic aging (body mass index [BMI], smoking, and saliva cell types) (Del Toro et al., 2024; Foster et al., 2023; Qi & Teschendorff, 2022). Adolescents' age at each time point was calculated from the caregiver‐reported date of birth and the date of the interview. Adolescents' biological sex and race/ethnicity were assessed via caregiver report at the initial assessment. Sex was coded as 1 = Female and 0 = Male while race was coded as 1 = Minority and 0 = White. At Time 1, caregivers indicated their annual household income on a 20‐point scale with response options ranging from less than $5,000 to more than $250,000. These reports were transformed into a percentage of the federal poverty line considering the household size and year of data collection (Harnett et al., 2019). The percentages were used as indicators of family income at Time 1 and standardized to facilitate model convergence in the main analysis.

At Time 3, BMI was computed from the average values of two height and weight measurements. If the two initial measurements were more than 0.5 cm or 0.2 kg apart, a third measurement was taken, and the two closest values were averaged. At Time 3, tobacco use was measured as a dichotomous indicator based on participants' self‐reports on whether they had smoked any cigarettes during the last year. Finally, cell type counts were measured in the Time 3 saliva samples using the reference‐based deconvolution method (Houseman et al., 2012). These scores included counts of CD4T, B‐cells, Monocytes, and Granular‐cells. The cell counts were standardized to facilitate model convergence in the main analysis (Schielzeth, 2010).

### Data analysis

#### Preliminary analyses

Before the main analyses, the percentage of cases with missing data and the percentage of missing data points were examined. Little's test of missing completely at random was conducted and follow‐up tests examined if cases with any missing data differed from cases with complete data on any of the variables included in the analyses using independent samples t‐tests for continuous variables and chi‐square tests for categorical variables. Additionally, descriptive statistics were obtained and bivariate correlations were examined among the main variables. All preliminary analyses were conducted in SPSS version 28 except for the Little's test which was conducted in STATA version 18.

#### Main analyses

The main analysis involved three longitudinal mediation path models predicting epigenetic aging on the GrimAge, DunedinPACE, and PhenoAge indicators. The analysis used maximum likelihood estimation with robust standard errors (MLR) due to non‐normal distributions of self‐esteem and psychological well‐being (Li, 2021). The models included paths from moral identity, empathy, and volunteering at Time 1 to self‐esteem and psychological well‐being at Time 2, as well as paths from self‐esteem and psychological well‐being at Time 2 to epigenetic aging at Time 3. In addition, the models included direct paths from moral identity, empathy, and volunteering at Time 1 to epigenetic aging at Time 3. Variables measured at the same time point were allowed to covary. Paths predicting self‐esteem and psychological well‐being at Time 2 were adjusted for self‐esteem and psychological well‐being at Time 1. All paths were adjusted for sex, race/ethnicity, and household income at Time 1. Effects on self‐esteem and psychological well‐being at Time 2 were also adjusted for chronological age at Time 2. Paths predicting epigenetic aging were also adjusted for BMI, tobacco use, and cell counts at Time 3. The indirect effects of moral identity, empathy, and volunteering on epigenetic aging through self‐esteem and psychological well‐being were tested with bias‐corrected bootstrapping using 10,000 bootstrap samples (Preacher & Hayes, 2008). Missing data were handled with full information maximum likelihood to retain the full sample size and minimize bias (Cham et al., 2017). The main analyses were conducted in Mplus version 8.

### Transparency and openness

This study with its hypotheses and methods was preregistered. The pre‐registration can be accessed in the Open Science Framework (OSF) repository using this anonymous view‐only link [https://osf.io/gtn9d/?view_only=2c5e7caee0a54292bb54f829ceb9a3e1]. The preregistration describes how the sample size was determined with all data exclusions. All measures and the coding of variables are described. De‐identified data and analysis codes are available in the Open Science Framework following this anonymous view‐only link [https://osf.io/w7r6t/?view_only=22ab71baba98422ba97df87afafeafb9].

## RESULTS

### Preliminary analyses

#### Missing data analysis

From the total number of 1,213 participants included in this study, 12% had missing data on at least one variable included in the analysis but only 2% of data points were missing from the total number of data points. Results from Little's MCAR test indicate that the data were not missing completely at random (MCAR) (χ^2^ [246] = 811.25, *p* < .001). Participants with missing data were older at Time 2 (*t*_[1,111]_ = 2.30, *p* = .022) and had lower psychological well‐being at Time 2 (*t*_[1,119]_ = −2.22, *p* = .027) and lower DunedinPACE at Time 3 (*t*_[153]_ = −2.61, *p* = .010). Participants with missing data did not differ from participants with complete data on any other variable included in the analysis. In sum, since individuals with missing data were older at Time 2, had lower psychological well‐being at Time 2, and had a lower DunedinPACE at Time 3, the mechanism of missingness is assumed to be that of missing at random (MAR).

#### Descriptive statistics and correlations

Descriptive statistics and results from the bivariate correlation analysis are reported in Table 1. The results showed that one in three adolescents engaged in volunteer work at Time 1. Higher moral identity was associated with higher empathy at Time 1 but neither moral identity nor empathy was associated with volunteering. Self‐esteem and psychological well‐being were positively intercorrelated at both Time 1 and Time 2. GrimAge, DunedinPACE, and PhenoAge acceleration were positively intercorrelated at Time 3. Both higher moral identity and higher empathy were associated with higher psychological well‐being at Time 1 and higher self‐esteem at Time 1 and Time 2. Volunteering was not associated with psychological well‐being or self‐esteem at any time point. Higher empathy at Time 1 was associated with lower GrimAge acceleration at Time 3 but unrelated to DunedinPACE or PhenoAge acceleration. Neither moral identity nor volunteering was associated with epigenetic aging on any clock. Higher self‐esteem at Time 1 was associated with slower epigenetic aging at Time 3 on both the GrimAge and DunedinPACE indicators but not on the PhenoAge clock. Self‐esteem at Time 2 and psychological well‐being at either Time 1 or Time 2 were not associated with epigenetic aging on any clock.

**TABLE 1:** Descriptive statistics and correlations among the Main variables.

	M (SD)	1.	2.	3.	4.	5.	6.	7.	8.	9.
1. Moral identity T1	4.39 (0.67)	1.00								
2. Empathy T1	2.71 (0.32)	0.38**	1.00							
3. Volunteering T1	33%	0.04	0.03	1.00						
4. Self‐esteem T1	3.63 (0.59)	0.20**	0.33**	−0.03	1.00					
5. Psychological WB T1	4.19 (0.79)	0.11**	0.15**	0.02	0.22**	1.00				
6. Self‐esteem T2	3.51 (0.58)	0.19**	0.10**	0.04	0.21**	0.13**	1.00			
7. Psychological WB T2	3.98 (0.73)	0.01	0.02	0.05	0.07*	0.31**	0.34**	1.00		
8. GrimAge T3	−0.51 (3.90)	−0.04	−0.09**	−0.04	−0.06*	0.01	−0.06	0.02	1.00	
9. DunedinPACE T3	0.00 (0.19)	0.01	−0.03	0.01	−0.07*	−0.05	−0.05	−0.03	0.36**	1.00
10. PhenoAge T3	−0.03 (5.70)	−0.04	−0.02	−0.02	−0.02	−0.01	−0.05	−0.03	0.32**	0.19**

*Note*: WB = Well‐being, T1 = Time 1, T2 = Time 2, T3 = Time 3.

* *p* < .05,

** *p* < .01.

### Main analyses

#### Test of H1

**FIGURE 1:**
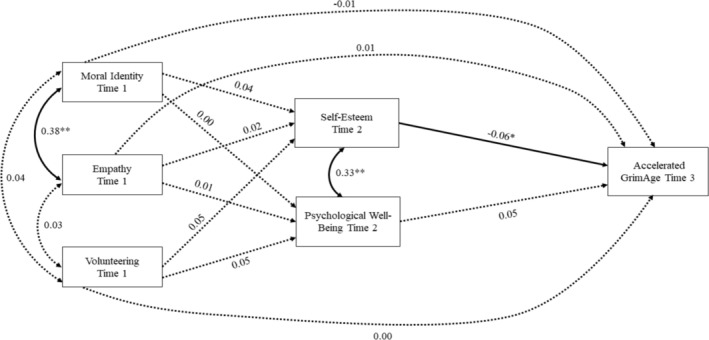
Mediation path model predicting GrimAge from the virtue variables through self‐esteem and psychological well‐being. *Note*: Standardized coefficients are shown. Solid lines indicate a significant effect. All paths adjusted for sex, race/ethnicity, and household income at time 1. Paths predicting self‐esteem and psychological well‐being were also adjusted for self‐esteem at time 1, psychological well‐being at time 1, and age at time 2. Paths predicting GrimAge were also adjusted for BMI, tobacco use, and cell counts at time 3. *p < .05; **p < .01.

The results from the first mediation path model predicting GrimAge acceleration are shown in Figure 1. The model had an excellent fit to the data (χ^2^_[17]_ = 38.23, *p* = .002; RMSEA = .03, CFI = .98). The results showed that moral identity, empathy, and volunteering had no unique effects on changes in self‐esteem or psychological well‐being from Time 1 to Time 2 and contrary to the hypothesis did not predict GrimAge acceleration at Time 3. However, higher self‐esteem at Time 2 uniquely predicted slower epigenetic aging on GrimAge at Time 3. Psychological well‐being did not predict GrimAge acceleration at Time 3.

**FIGURE 2:**
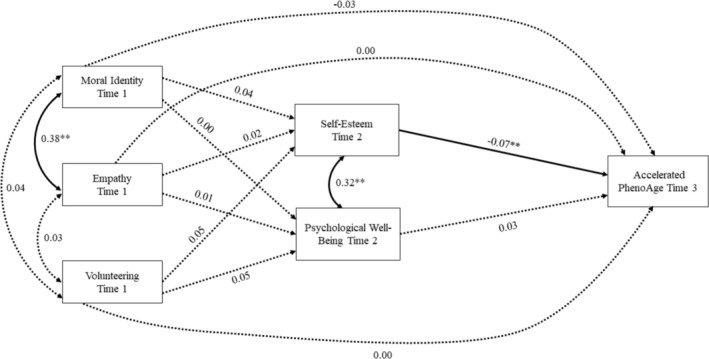
Mediation path model predicting PhenoAge from the virtue variables through self‐esteem and psychological well‐being. *Note*: Standardized coefficients are shown. Solid lines indicate a significant effect. All paths adjusted for sex, race/ethnicity, and household income at time 1. Paths predicting self‐esteem and psychological well‐being were also adjusted for self‐esteem at time 1, psychological well‐being at time 1, and age at time 2. Paths predicting PhenoAge were also adjusted for BMI, tobacco use, and cell counts at time 3. *p < .05; **p < .01.

The results from the second mediation path model predicting PhenoAge acceleration replicated the results from the first Model (see Figure 2). The model had an excellent fit to the data (χ^2^_[17]_ = 48.66, *p* < .001; RMSEA = .04, CFI = .96). As in the first model, the results showed no unique effects of moral identity, empathy, or volunteering on changes in self‐esteem or psychological well‐being from Time 1 to Time 2. Moreover, contrary to the hypothesis, moral identity, empathy, and volunteering did not predict PhenoAge acceleration at Time 3. However, higher self‐esteem at Time 2 uniquely predicted slower epigenetic aging on PhenoAge at Time 3, while psychological well‐being did not affect PhenoAge acceleration at Time 3.

**FIGURE 3:**
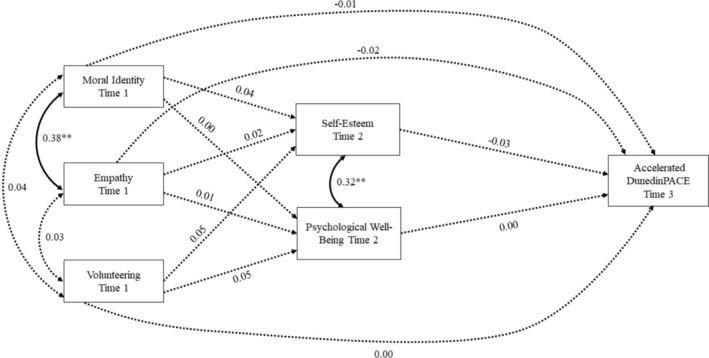
Mediation path model predicting DunedinPACE from the virtue variables through self‐esteem and psychological well‐being. *Note*: Standardized coefficients are shown. Solid lines indicate a significant effect. All paths adjusted for sex, race/ethnicity, and household income at time 1. Paths predicting self‐esteem and psychological well‐being were also adjusted for self‐esteem at time 1, psychological well‐being at time 1, and age at time 2. Paths predicting DunedinPACE were also adjusted for BMI, tobacco use, and cell counts at time 3. *p < .05; **p < .01.

The results from the third mediation path model predicting epigenetic aging on DunedinPACE are shown in Figure 3. As the previous models, the model had an excellent fit to the data (χ^2^_[17]_ = 48.41, *p* < .001; RMSEA = .04, CFI = .97). As in the previous two models, moral identity, empathy, and volunteering had no unique effects on changes in self‐esteem or psychological well‐being from Time 1 to Time 2. Moreover, contrary to the hypothesis, moral identity, empathy, and volunteering did not predict DunedinPACE at Time 3. In contrast to the prior models involving GrimAge or PhenoAge, neither self‐esteem nor psychological well‐being at Time 2 predicted epigenetic aging at Time 3.

#### Test of H2

Results from the bootstrap analyses of indirect effects from all models are presented in Table 2. Contrary to the hypothesis, the results showed no indirect effects of moral identity, empathy, or volunteering on GrimAge acceleration through either self‐esteem or psychological well‐being. Similarly, contrary to the hypothesis, results from the bootstrap analyses showed no indirect effects of moral identity, empathy, or volunteering on PhenoAge acceleration through either self‐esteem or psychological well‐being. Finally, contrary to the hypothesis, results from the bootstrap analysis showed no indirect effects of moral identity, empathy, or volunteering on DunedinPACE through either self‐esteem or psychological well‐being.

**TABLE 2:** Indirect effects of the virtue variables on epigenetic aging through self‐esteem and psychological well‐being.

Indirect effects/outcomes	GrimAge T3	PhenoAge T3	DunedinPACE T3
	Beta [95% CI]	Beta [95% CI]	Beta [95% CI]
Moral identity T1 ‐ > self‐esteem T2	−0.002 [−0.010, 0.001]	−0.003 [−0.012, 0.002]	−0.001 [−0.007, 0.001]
Moral identity T1 ‐ > psychological well‐being T2	0.000 [−0.004, 0.003]	0.000 [−0.003, 0.002]	0.000 [−0.001, 0.001]
Empathy T1 ‐ > self‐esteem T2	−0.001 [−0.008, 0.002]	0.002 [−0.008, 0.003]	−0.001 [−0.006, 0.001]
Empathy T1 ‐ > psychological well‐being T2	0.001 [−0.002, 0.006]	0.000 [−0.001, 0.005]	0.000 [−0.002, 0.002]
Volunteering T1 ‐ > self‐esteem T2	−0.003 [−0.009, 0.000]	−0.003 [−0.010, 0.000]	−0.001 [−0.006, 0.000]
Volunteering T1 ‐ > psychological well‐being T2	0.002 [0.000, 0.008]	0.001 [−0.001, 0.006]	0.000 [−0.002, 0.002]

*Note*: T1 – Time 1, T2 – Time 2, T3 – Time 3. Standardized coefficients and corresponding confidence intervals are shown.

## DISCUSSION

The assertion that individuals should strive to live a virtuous life for their own well‐being is an ancient one (Aristotle, ca. 350 BCE/Bartlett & Collins, 2011). However, the potential health benefits of virtues during adolescence have remained largely unexplored. Thus, the current study sought to extend our understanding of the relationships between three key dimensions of virtue in early adolescence and epigenetic aging in late adolescence. Additionally, considering the possible effects of virtues on self‐esteem and psychological well‐being, these constructs were examined as potential mediators linking moral identity, empathy, and volunteering with epigenetic aging (Brown et al., 2012; Hardy et al., 2013; Hui et al., 2020; Supervía et al., 2023; Vinayak & Judge, 2018).

The results showed that moral identity, empathy, and volunteering in early adolescence did not predict self‐esteem or psychological well‐being in middle adolescence or epigenetic aging in late adolescence after adjusting for previous self‐esteem, psychological well‐being, and other covariates. Additionally, self‐esteem and psychological well‐being did not mediate the relationship between any of the virtues and epigenetic aging. Nevertheless, self‐esteem in middle adolescence predicted lower epigenetic aging for the GrimAge and PhenoAge epigenetic clocks in late adolescence. Additionally, correlation analyses showed an association between higher empathy in early adolescence and lower epigenetic aging on the GrimAge clock in late adolescence, consistent with previous empirical findings (Dobewall et al., 2023; Goering, Moore, et al., 2024).

The lack of significant links between the virtues and outcomes may be due to the developmental timing of virtue assessment coupled with the long gaps between the time points (3 and 6 years). In early adolescence, moral identity is still in its formative stages (Krettenauer & Hertz, 2015) and may not yet be fully developed to impact psychological well‐being and self‐esteem, especially across the span of three years. At this stage, social identity may take precedence over moral identity (Tanti et al., 2011), further lessening the potential role of moral identity in self‐esteem and psychological well‐being. Moreover, early adolescents typically have limited opportunities to volunteer, and even less so if they come from low SES backgrounds (Lichter et al., 2002). Research also suggests that feelings of social responsibility decrease from age 9 to age 16 before stabilizing in late adolescence (Wray‐Lake et al., 2016), which may also reduce the likelihood of volunteering during early adolescence. Similarly, there is a temporal decline in empathy during early adolescence followed by a steady increase from middle adolescence into early adulthood (Van der Graaff et al., 2014).

Relatedly, early adolescence is characterized by substantial neural, cognitive, and social–emotional changes, as well as heightened stress related to pubertal development, school transitions, and evolving family and peer relationships (Holder & Blaustein, 2014; Somerville, 2013; Stroud et al., 2009). This heightened stress may reduce cognitive resources available for virtuous cognitions and weaken their potential links to self‐esteem, psychological well‐being, and epigenetic aging. Thus, the combination of a still‐developing moral identity, reduced empathy, few volunteering opportunities, and elevated stress during early adolescence may explain the lack of significant associations with self‐esteem, psychological well‐being, and epigenetic aging in later adolescence. Future studies should examine these relationships in middle to late adolescence utilizing shorter time periods between assessments.

The present results also need to be interpreted in a cultural context, such as individualistic vs collectivistic orientation. Given that this study was conducted in the U.S., which is considered to be a highly individualistic society (Bazzi et al., 2020; Minkov & Kaasa, 2022), different results may be obtained in studies conducted in more collectivistic cultures. For example, moral identity, empathy, and volunteering may be more strongly related to slower epigenetic aging in more collectivistic cultures due to greater societal value and positive reinforcement placed on these behaviors. Thus, examining these questions in cross‐cultural replication studies will present an interesting avenue for future research.

Finally, the novel finding that self‐esteem uniquely predicts lower accelerated epigenetic aging on the GrimeAge and PhenoAge Clocks is intriguing. It suggests that adolescents who hold themselves in high regard may experience slower biological aging compared to their peers. This could indicate that higher self‐esteem helps reduce the likelihood of stress‐inducing situations and mitigate stress levels, thereby contributing to slower epigenetic aging. Indeed, higher self‐esteem has been linked to better‐coping strategies (Li et al., 2023), which may help buffer the effects of stress on the body. Furthermore, self‐esteem is more strongly related to happiness in late adolescence than other factors, such as parental rearing styles and personality traits (Furnham & Cheng, 2000). These findings highlight the potential health benefits of self‐esteem during adolescence. Interestingly, self‐esteem did not uniquely predict epigenetic aging on the DunedinPACE epigenetic clock, underscoring the importance of utilizing multiple epigenetic clocks in epigenetic studies as they cover different aspects of biological aging and health (Oblak et al., 2021).

### Implications

The observed link between higher self‐esteem in middle adolescence and lower epigenetic aging in late adolescence carries important implications. For example, interventions focused on improving adolescents' health may want to incorporate strategies to enhance self‐esteem. Moreover, these results suggest that youth with lower self‐esteem could benefit from targeted interventions to mitigate accelerated epigenetic aging, potentially reducing their risk of later health problems. Future research should explore whether the positive impact of self‐esteem on epigenetic aging persists into adulthood. Finally, although the hypothesized relationships between virtues and epigenetic aging were not supported, this study lays an important foundation for future research exploring how virtues may affect self‐esteem, psychological well‐being, and epigenetic aging during adolescence.

### Strengths, limitations, and future directions

The current study has several notable strengths, including the longitudinal design, which allowed for testing the temporal relationships between virtues, self‐esteem, and psychological well‐being. Moreover, the use of virtues that capture three dimensions of morality (cognitive, emotional, and behavioral) is also a strength. Another strength is the diversity of the community‐based sample, which enhances the generalizability of the findings. However, several limitations should be considered. First, this study was not able to adjust for prior levels of epigenetic aging when it was predicted from prior virtues, self‐esteem, and psychological well‐being, limiting the ability to establish temporal precedence. Thus, future studies should collect epigenetic data over multiple time periods to further our understanding of the temporal relationships between virtues, self‐esteem, psychological well‐being, and epigenetic aging. Additionally, the use of saliva as the source for DNA methylation has limitations as prior research suggests that DNA methylation extracted from other tissue samples such as blood tends to result in larger effect sizes (Raffington et al., 2023). However, given that the present study consists of adolescents, utilizing saliva for the basis of DNA methylation extraction presents an easier method of collection than collecting blood samples as providing a saliva sample is more readily accepted than blood by the target population. This is also in line with prior research that is conducted with children and adolescents (Raffington, 2024; Raffington et al., 2024). Nonetheless, replicating the current study utilizing DNA methylation from other tissues would be informative.

Second, the study used a less established measure of moral identity. This limits our ability to directly compare the present results with prior empirical findings. However, it is likely that the moral identity measure used in the present study is highly correlated with the internalizing subscale of the Self‐Importance of Moral Identity Scale (Aquino & Reed, 2002) as they both assess the degree to which values of helpfulness, fairness, honesty, integrity, altruism, and work ethic are integrated into one's identity. Interestingly, the internalizing subscale of the Self‐Importance of Moral Identity Scale is often not combined with the symbolization subscale, which measures outward moral behavior in empirical research assessing moral identity (see Goering, Espinoza, et al., 2024; Hertz & Krettenauer, 2016). Thus, it may be possible to compare the present results with other findings that utilized only the internalization subscale, albeit with caution. Nonetheless, future studies would benefit from using more widely utilized measures of moral identity such as the Self‐Importance of Moral Identity scale (Aquino & Reed, 2002) to validate the present results.

Next, the single volunteering item only assessed whether youth participated in volunteering activities, but did not capture the frequency or intensity of volunteering. While this methodology limited insights into the potential effects of volunteering frequency and intensity on our results, this approach is consistent with prior empirical work that assessed volunteering in early adolescence (Lanza et al., 2023) as volunteering behaviors are typically not performed frequently at this age (Lichter et al., 2002). However, future research should use more detailed measures of volunteering, especially during middle and late adolescence as it becomes a more frequent behavior.

Additionally, the three virtues were only assessed in early adolescence, limiting the ability to test whether the results would differ if these constructs were measured in middle adolescence or late adolescence. Future research should replicate these results by assessing moral identity, empathy, and volunteering across multiple stages of adolescence to better understand their developmental trajectories and long‐term impacts on self‐esteem, psychological well‐being, and epigenetic aging. Additionally, the time intervals between assessments were three years apart which may have obscured short‐term developmental changes and dynamic relationships between virtues, self‐esteem, psychological well‐being, and epigenetic aging. Given that early adolescence is a critical time for the cultivation of virtues, which may include fluctuations in moral identity, empathy, and volunteering during that time (Krettenauer & Hertz, 2015; U. S Census Bureau, 2021; Van der Graaff et al., 2014), links between virtues and psychological mediators and epigenetic aging may be more immediate. Thus, future research would benefit from shorter periods between assessments.

Finally, while the present study's focus on the three dimensions of virtue (cognition, emotion, and behavior) is a strength, other virtues, such as humility, gratitude, and resilience, may also be related to epigenetic aging. One potential mechanism for how humility, gratitude, and resilience may be linked to slower epigenetic aging is emotion regulation. For example, individuals with higher levels of humility, gratitude, or resilience may cultivate better emotion regulation abilities (Valdez & Datu, 2021; Yu et al., 2021) that then reduce stress (Hartley & Phelps, 2010) and lead to slower epigenetic aging. Thus, future research should expand the scope of virtues beyond those examined in this study. Finally, another interesting avenue of future research would be examining self‐esteem and psychological well‐being as moderators of the relationship between virtues and epigenetic aging at various times in adolescence and adulthood. Future research would also benefit from explicitly measuring and incorporating cultural constructs such as collectivistic vs. individualistic orientation. These orientations may influence the expression and impact of virtues, potentially moderating the pathways between moral identity, psychological well‐being, and epigenetic aging.

## CONCLUSION

The current study indicates no relationship between virtues in early adolescence and epigenetic aging in late adolescence, nor does it support the hypothesis that this relationship is mediated by self‐esteem and psychological well‐being in middle adolescence. However, more research is needed to confirm these findings. Examining moral identity, empathy, and volunteering at multiple time points throughout adolescence will provide deeper insights into their potential roles in epigenetic aging and the roles of self‐esteem and psychological well‐being as potential mediators. Additionally, the novel finding that self‐esteem uniquely predicts a lower rate of epigenetic aging suggests that confidence in one's own abilities may contribute to slower biological aging relative to same‐age peers. These findings underscore the potential health benefits of fostering self‐esteem during adolescence. Nevertheless, at least for the period of early adolescence, being virtuous may be its own reward after all.

## CONFLICT OF INTEREST STATEMENT

The authors declare they have no conflict of interest to disclose.

## ETHICS STATEMENT

This study was approved by the institutional review board. Written informed consent was obtained from adult participants and legal guardians. Written assent was obtained from adolescents.

## Data Availability

The data that support the findings of this study are openly available in Open Science Framework at https://osf.io/w7r6t/?view_only=22ab71baba98422ba97df87afafeafb9.
